# Congenital Heart Disease: The State-of-the-Art on Its Pharmacological Therapeutics

**DOI:** 10.3390/jcdd9070201

**Published:** 2022-06-26

**Authors:** Carlos Daniel Varela-Chinchilla, Daniela Edith Sánchez-Mejía, Plinio A. Trinidad-Calderón

**Affiliations:** 1 Tecnológico de Monterrey, School of Medicine and Health Sciences, Ave. Ignacio Morones Prieto 3000 Pte., Col. Los Doctores, Monterrey 64710, N.L., Mexico; carlos.varela.ch@gmail.com (C.D.V.-C.); a01365669@tec.mx (D.E.S.-M.); 2 Tecnológico de Monterrey, Escuela de Ingeniería y Ciencias, Ave. Eugenio Garza Sada 2501, Monterrey 64849, N.L., Mexico

**Keywords:** CHD, congenital, heart, disease, pharmacological, treatment, state-of-the-art

## Abstract

Congenital heart disease is one of the most common causes of death derived from malformations. Historically, its treatment has depended on timely diagnosis and early pharmacological and surgical interventions. Survival rates for patients with this disease have increased, primarily due to advancements in therapeutic choices, but mortality remains high. Since this disease is a time-sensitive pathology, pharmacological interventions are needed to improve clinical outcomes. Therefore, we analyzed the applications, dosage, and side effects of drugs currently used for treating congenital heart disease. Angiotensin-converting enzyme inhibitors, angiotensin receptor blockers, beta-blockers, and potassium-sparing diuretics have shown a mortality benefit in most patients. Other therapies, such as endothelin receptor antagonists, phosphodiesterase-5 inhibitors, prostaglandins, and soluble guanylyl cyclase stimulators, have benefited patients with pulmonary artery hypertension. Likewise, the adjunctive symptomatic treatment of these patients has further improved the outcomes, since antiarrhythmics, digoxin, and non-steroidal anti-inflammatory drugs have shown their benefits in these cases. Conclusively, these drugs also carry the risk of troublesome adverse effects, such as electrolyte imbalances and hemodynamic compromise. However, their benefits for survival, symptom improvement, and stabilization outweigh the possible complications from their use. Thus, cases must be assessed individually to accurately identify interventions that would be most beneficial for patients.

## 1. Introduction

Congenital heart disease (CHD) describes a set of cardiac structural malformations resulting from alterations during embryonic organogenesis [1]. Currently, CHD is recognized as the leading cause of mortality from birth defects [2]. Worldwide, it affects approximately 10% of all births [3]. Furthermore, about 20–25% of CHDs are considered critical because they require medical and surgical care to survive [4].

Survival rates for CHD patients have improved in developed regions of the world, reaching even 90% [5,6,7], leading to an increase in the number of adult CHD patients [6,8]. However, in developing regions, CHD is still associated with high mortality [4,6], with an average of 4.9 deaths per 100,000 cases compared with 1.2 deaths per 100,000 cases in developed regions [2].

Due to this incidence, early intervention for CHDs is considered essential for pediatric patients [9], since this condition must be addressed with a combination of catheter-based, pharmacological, and surgical treatment [10]. Moreover, many of the pharmacological interventions have been shown to reduce mortality in CHD patients, thus emphasizing the importance of their application [11,12,13,14].

Currently, pharmacological therapy for patients with CHD is largely empirical, due to the pressing need to prolong and improve the quality of life for these patients [15]. Moreover, innovation is needed in the field of drug therapies for CHD, as well as for recommendations on rational management and use of latest generation drugs [16,17,18].

Therefore, herein we present a comprehensive review of the state-of-the-art of drugs for the treatment of patients with CHD. Furthermore, we show the spectrum of mechanisms of action and the indications, dosing regimens, and adverse effects/contraindications of each of the addressed drugs. Finally, we discuss the most recent clinical trials testing different drugs for CHD treatment.

### Methodology for Literature Research

We searched and retrieved Google Scholar and Scopus databases for the keywords adult, clinical, congenital, chd, disease, drug, heart, pediatric, pharmacological, therapy, treatment, and trial, inspired by PRISMA guidelines (Figure S1) [19]. Both original and review articles were selected as relevant if they were published from 2017 onwards. Those articles containing the keywords dental, device, catheter, reflux, repair, regenerative, valve, and ultrasound were discarded.

## 2. Drugs for CHD Treatment

**Figure 1:**
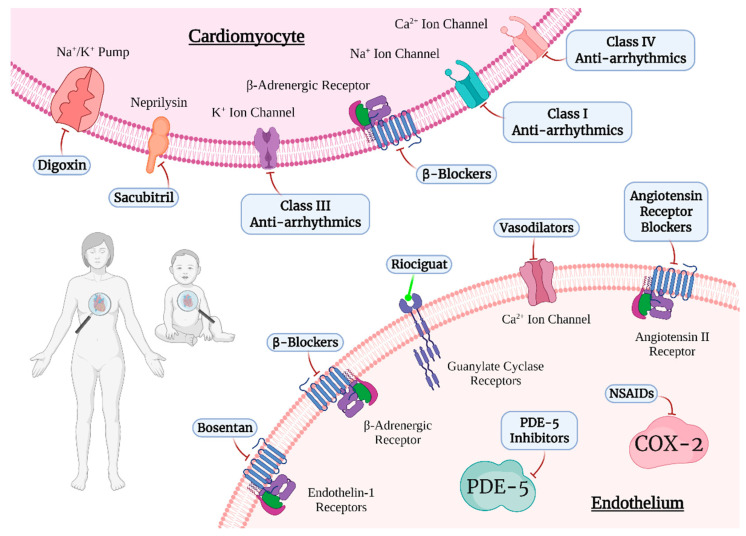
Drugs for CHD treatment with molecular targets on cardiomyocytes and endothelial cells.

To date, the pharmacological treatment of pediatric CHD has been extrapolated from the cornerstones of cardiovascular treatment in adults [20]. Recent studies have shown that patients with CHD exhibit pathological neurohormonal activation and cardiac remodeling similar to acquired heart disease [21]. Therefore, we analyzed drugs with both known and potential benefits for patients with CHD in this section (Figure 1).

### 2.1. Beta-Blockers

The blockade of beta-adrenergic receptors in the heart decreases cardiac output, myocardial strain, oxygen demand, heart rate, contractility, and blood pressure, and promotes coronary vasodilation (Figure 1) [22,23].

Recently, some studies have demonstrated that CHDs also affect myocardial cell division and cytokinesis, i.e., phenomena that can be prevented with beta-blockers [24]. In particular, beta-blockers have a wide array of labeled and off-label cardiovascular indications (Table 1) [25]. Over time, three generations of them have been marketed for treating hypertension and heart failure [26].

Precisely, first-generation beta-blockers are non-selective against both β_1_ and β_2_ receptors (e.g., propranolol), second-generation beta-blockers are more cardio-selective (β_2_) (e.g., atenolol), and third-generation blockers vary selectivity for β_1_-receptors as well as vasodilatory properties (e.g., nebivolol) [33]. Specifically, the third-generation beta-blocker carvedilol contains a 2-methoxy-phenyl-ethyl residue at the allopathic nitrogen that is responsible for its vasodilating properties [34].

Furthermore, these molecules reduce cardiac remodeling, the incidence of ventricular arrhythmias, and the risk of sudden cardiac death, and also prevent arrhythmias by modulating the cardiac conduction system [35,36]. Nonetheless, clinical studies regarding the use of beta-blockers, specifically the third-generation beta-blocker carvedilol, did not show any treatment effect on clinical heart failure outcomes, even though many authors claim the dosage was too low for an effect to take place [37].

Specifically, a population pharmacokinetics study demonstrated that pediatric patients had to receive up to four times the dosage recommended for adults to achieve a comparable bioavailability in blood [28]. Recently, the use of the highly cardio-selective, long-acting beta-blocker bisoprolol has been proposed for pediatric heart failure, as it has a dual mechanism of the β1-receptor blockade and endothelial nitric oxide production and may decrease myocardial fibrosis and lower systemic vascular resistance [27]. Additionally, propranolol is currently the treatment of choice in heart failure caused by pediatric hypertrophic cardiomyopathy [38].

### 2.2. Inhibitors of Renin–Angiotensin–Aldosterone System

The blockade of the renin-angiotensin-aldosterone system (RAAS) has shown both cardioprotective and nephroprotective characteristics—e.g., ACE inhibitors (ACEIs) and angiotensin receptor blockers (ARBs) have proven to be effective in hypertension and heart failure of any cause [39]. RAAS involves an intricate relation between hormones which ultimately results in sodium and water retention in nephrons [40], thus physiologically maintaining systemic blood pressure [41]. In this regard, the chronic activation of RAAS induces hypertension and fibrotic changes in the kidney [42].

Thus, we explored both ACEIs and ARBs as pharmaceutical options for CHD treatment in this subsection.

#### 2.2.1. Angiotensin-Converting Enzyme Inhibitors

ACEIs decrease the adrenergic activity and RAAS activation [43], thus reducing symptoms related to increased blood pressure and sympathetic tone, reducing the progression of heart failure, limiting hospitalizations, and improving survival [14]. They prevent cardiac remodeling by inhibiting the production of extracellular matrix and reducing the pro-inflammatory effect of cytokines on the vascular endothelium [36]. This is particularly useful in patients with heart failure and low ejection fraction [22].

Captopril, one of the most widely used ACEIs, was introduced as a safe and effective drug for hypertension and congestive heart failure in 1981 [44]. Its efficacy in pediatrics has been demonstrated by a reduction in left ventricular overload and hypertrophy in children [45]. Currently, this drug is recommended for newborns and infants, while lisinopril and enalapril are recommended for older children (Table 2) [46].

In 2013, the use of ACEIs was approved for the treatment of pediatric heart failure, regardless of etiology [46]. However, its effects have not been thoroughly studied [50]. To date, there is a class I recommendation for patients with left ventricular dysfunction for the use of ACEIs, as well as a class IIa recommendation for asymptomatic patients [14].

Some studies have shown that adults with heart failure and children with dilated cardiomyopathy or systolic ventricular function treated with ACEIs had better survival at a one- and two-year follow-up compared with those treated with digoxin and potassium diuretics, such as spironolactone (Section 2.5.3 and Section 2.3.2, respectively) [13,14].

Moreover, studies have shown that there is clinical improvement in pediatric patients with left-to-right shunts with heart failure, but not in those with heart failure caused by pressure overload lesions [51].

#### 2.2.2. Angiotensin Receptor Blockers

Angiotensin receptors were initially discovered in blood vessels and adrenal glomerulosa [52]. ARBs, such as valsartan and losartan, directly inhibit angiotensin II receptors [53]. Furthermore, the inhibition of the final phase of the RAAS system by ARBs provides a more efficient blockade of cardiovascular effects of angiotensin II with fewer side effects than ACEIs (Table 3) [49].

Specifically, their primary indication is for children who are intolerant to ACEIs [14]. Interestingly, a recent double-blind, randomized, clinical trial in children aged between 1–16 years showed that treatment with valsartan improved clinical, electrocardiographic, and echocardiographic characteristics of patients with heart failure due to a CHD with left-to-right shunt [55].

Interestingly, adult patients with heart failure due to CHD treated with ARBs showed a decrease in systolic blood pressure and tricuspid regurgitation, as well as an increase in exercise duration in those with great vessel transposition [57]. Additionally, ARBs have been shown to improve left ventricular ejection fraction in adults with heart failure [14].

By comparison, ARBs have the particular advantage of once-daily administration, which improves drug compliance [49]. However, studies have shown no significant changes in the mean ejection fraction, peak ventilatory oxygen equivalent, or ventricular dimensions in both children and adult patients with corrected tetralogy of Fallot, systemic right ventricle, and hypoplastic left heart syndrome treated with these drugs [58]. Moreover, the only randomized clinical trial comparing ARBs (losartan 25 mg/d) with ACEIs (lisinopril 5 mg/d) was performed in patients with Duchenne muscular dystrophy, demonstrating a significant improvement in left ventricular ejection fraction sustained at 1 year, but without a significant difference between both groups [14].

### 2.3. Diuretics

**Figure 2:**
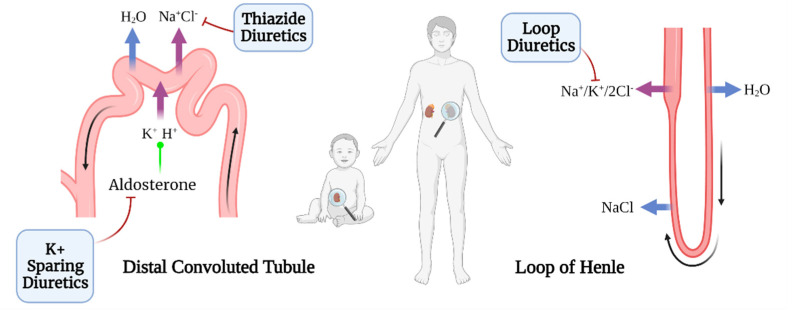
Diuretics targeting different regions of the kidney (nephron) for CHD treatment.

Diuretics have been a preferred therapy for cardiovascular diseases that are widespread in recent decades [59]. They serve as the first line of treatment for children with congestive heart failure, regardless of its cause [60]. Here, we accurately analyzed loop, thiazide, and potassium-sparing diuretics (Figure 2). Notwithstanding, carbonic anhydrase inhibitors, the remaining class of diuretic, have shown no benefit in treating volume overload, and their wide range of side effects makes them unsuitable for clinical therapy [61].

#### 2.3.1. Loop Diuretics

These drugs were initially proposed along with digitalis for pediatric acute heart failure [62] and are considered first-line therapy for congestive heart failure [43]. Their successful application was achieved until 1971, when furosemide, the most common loop diuretic [63], proved to be a quick and safe alternative for fluid overload in children (Table 4) [64]. These drugs target the reabsorption of chloride and sodium by inhibiting the Na^+^/K^+^/2Cl^−^ cotransporter in the thick ascending limb of the loop of Henle [14].

Due to their safety profile and extensive clinical experience, metabolically neutral loop diuretics are preferred in adult patients with a right-to-left shunt or Eisenmenger syndrome [68]. However, the most appropriate dose and frequency of administration remain to be determined [14].

Furthermore, studies have determined that in children hospitalized with acute decompensated heart failure, a decreased diuretic response was associated with increased mortality, longer inpatient stay, and worse prognosis [60]. However, recent evidence has shown that the use of continuous diuretics may be beneficial to neonates, especially after cardiac bypass, as a continuous infusion of furosemide (0.1 mg/kg/h) had a higher diuretic response and a higher likelihood of achieving a negative balance than an intermittent bolus of 1 mg/kg IV q4h [14].

#### 2.3.2. Thiazide Diuretics

These diuretics cause a natriuretic effect and a decrease in extracellular volume, venous return, cardiac output, and peripheral vascular resistance at high doses by targeting the reabsorption of sodium in the distal renal tubules [69]. Furthermore, both extracellular volume and cardiac output return to normal when administered chronically, but peripheral vascular resistance continues to decrease [70].

Clinically, thiazide diuretics can be used synergistically with furosemide in children with refractory volume overload in the setting of congestive heart failure (Table 5) [14]. In 1957, the first thiazide diuretic, chlorothiazide, entered the market as a safe and effective oral diuretic, followed by hydrochlorothiazide, a molecule 10–15 times more potent, one year later [71]. Both molecules act on the distal convoluted tubule inhibiting the sodium chloride cotransporter (Figure 2) [69].

#### 2.3.3. Potassium-Sparing Diuretics (Mineralocorticoid Antagonists)

These diuretics bind to the mineralocorticoid receptor and antagonize aldosterone, resulting in the inhibition of both sodium reabsorption and potassium excretion (Figure 2) [72]. To date, the most potent potassium-sparing diuretic with improved intestinal absorption is spironolactone [71], a drug that reduces mortality by 30% in adults with CHD (Table 6) [46].

Both spironolactone and eplerenone, another potassium-sparing diuretic [71], prevent myocardial fibrosis and excessive catecholamine secretion [22]. Furthermore, eplerenone has fewer adverse effects than spironolactone [75]. Additionally, recent studies in adults have demonstrated that this drug downregulates osteopontin, a hormone associated with cardiac remodeling and fibrosis, resulting in additional benefits [76]. Moreover, the combination of spironolactone (0.5–1 mg/kg) with lisinopril (0.1–0.2 mg/kg/d) and bisoprolol (0.1–0.2 mg/kg/d) is beneficial for pediatric patients as it reduces systemic vascular resistance and may reduce cardiac fibrosis [77].

Regarding patients post-operation with the Fontan procedure, protein-losing enteropathy is a common complication [78]. Spironolactone improves cardiac and endothelial cell function and reduces inflammation in the presence of this condition [78,79]. Nonetheless, in a 4-week trial study with 12 pediatric patients with Fontan-type physiology and heart failure, the administration of spironolactone was associated with a significant reduction in interleukin-1b, but no other significant changes were seen [80].

### 2.4. Vasodilators

In 1980, Furchgott and Zawadzki discovered that acetylcholine and bradykinin stimulated endothelium to produce a vasodilating substance called the endothelium-1-derived relaxing factor [81]. Subsequently, this factor was identified as nitric oxide (NO) [82]. NO, which is produced in the myocardium [83], is responsible for inducing vasodilation, as well as positive inotropic and lusitropic effects in the heart [84] through SGC-mediated cGMP production [85].

Hence, we discussed the role of different vasodilating drugs in CHD treatment in this subsection.

#### 2.4.1. Endothelin-1 Receptor Antagonists

Endothelin-1 is a peptide implicated in hypertension, chronic kidney disease, and impaired lung function, in addition to inducing cardiac remodeling, increased atrial diameter, and left ventricular mass [86]. ERAs, such as bosentan and ambrisentan (Table 7), have shown favorable results in reducing the deleterious effects of endothelin-1 [87]. Consequently, they improve the survival of adult patients, particularly those with symptomatic pulmonary arterial hypertension associated with CHDs [88].

In particular, bosentan is an antagonist of endothelin A (ET_A_) and B (ET_b_) receptors [93], which has been used to reduce pulmonary vascular resistance since 2004 [94,95]. It is also indicated for adults with Eisenmenger syndrome [91]. It has also been shown to delay the need for transplants and increase the quality of life in the meantime [96].

The pharmacokinetics of bosentan in pediatric pulmonary arterial hypertension and healthy adults are similar [92]. Studies have shown that the exposure plateau for bosentan is reached at a dose of 2 mg/kg twice daily, making the adequate dose up to 4 mg/kg [97]. Currently, incremental treatment with bosentan along with sildenafil has been shown to improve pulmonary and systemic vascular resistance in a study with patients ranging from 12 to 53 years with CHD and pulmonary arterial hypertension [98]. However, macitentan, an analogous-to-bosentan pulmonary vasodilator ERA [94], improved mortality and morbidity in a placebo-controlled trial of bosentan [99].

#### 2.4.2. Phosphodiesterase Inhibitors

Phosphodiesterase-5 (PDE-5) is the enzyme that catabolizes cGMP to its inactive metabolite [100]. Its inhibition causes intracellular accumulation of cGMP, the eventual induction of smooth muscle relaxation, and a decrease in oxygen consumption and inotropy [101].

Studies have found that PDE-5 inhibitors confer significant benefits against death and hospitalization in patients older than 18 years with reduced left ventricular ejection fraction [85]. Particularly, sildenafil and tadalafil, both inhibitors of PDE-5 (Table 8), are the basis of pulmonary arterial hypertension treatment due to their vasodilatory effects [102], along with diuretics to control right ventricular overload [103].

They are also the treatment choice for pulmonary arterial hypertension resistant to NO [105] and have also been associated with increased survival in adolescent and adult patients with Eisenmenger syndrome [12]. In recent decades, milrinone, a phosphodiesterase III inhibitor, has become an alternative as it increases myocardial contractility while also decreasing both systemic and pulmonary vascular resistance, with a greater reduction in the post-capillary wedge pressure than dobutamine [75].

#### 2.4.3. Prostaglandins (PGs)

Ductus-dependent CHDs require ductal patency to avoid the impairment of end-organ perfusion and hypoxia due to inadequate pulmonary flow, as well as intracardiac mixing [106]. Derived from arachidonic acid, PGs are endogenous autacoid lipids involved in the body’s inflammatory response [107]. In 1973, Coceany and Olley demonstrated the efficacy of PG E1 and E2 in relaxing the ductus arteriosus [108]. Both molecules were first used in children in 1975 and were further approved by the Food and Drug Administration in 1981 [108,109].

The decision to initiate treatment with PG is based on the antenatal diagnosis of a ductus-dependent CHD or clinical findings, such as cyanosis or absence of femoral pulses, with or without acidosis [110]. PGE1 can be administered by continuous infusion to stabilize the infant’s condition before surgery [111]. Early treatment with PG E1 is associated with lower rates of morbidity and mortality (Table 9) [11].

Similarly, epoprostenol and intravenous prostacyclin have been shown to increase cardiac index and decrease in the NYHA class of symptoms [12]. In particular, this last drug binds to endothelial prostacyclin receptors, causing an increase in cAMP, resulting in vasodilation [12].

#### 2.4.4. Stimulators of Soluble Guanylate Cyclase

The discovery and elucidation of soluble guanylate cyclase (sGC) reporting dates to 1998 [114]. sGC stimulators increase NO production in various tissues [115]. The resulting increase in cGMP derived from NO stimulation also inhibits vascular remodeling [116]. Additional benefits of sGC include improved pulmonary vascular resistance, WHO functional class, and reduced levels of N-terminal pro-brain natriuretic peptide [12].

Among the sGC stimulators, riociguat was approved by the FDA for treating pulmonary arterial hypertension in October 2013 (Table 10) [117,118]. It was originally intended for treating pulmonary arterial hypertension in adults associated with CHD [119]. Recently, riociguat has been shown to significantly reduce pulmonary vascular resistance and increase cardiac index in patients with CHDs and pulmonary arterial hypertension [120]. Additionally, it showed improvement in a 6 min walking distance, exercise capacity, and functional capacity at 2 years [12].

### 2.5. Other Pharmacological Options for CHD Treatment

Adjunctive medications with known benefits, such as arrhythmia prevention [121], symptom reduction [122], mitigation of neurohormonal activation [50], and closure of the patent ductus arteriosus in treating CHDs [123], are reported for angiotensin receptor-neprilysin inhibitors (ARNIs) [103,124], antiarrhythmics [125], digoxin [126], and non-steroidal anti-inflammatory drugs (NSAIDs) [127].

Therefore, we studied these additional pharmacological options in this subsection.

#### 2.5.1. Angiotensin Receptor-Neprilysin Inhibitors

Neprilysin, first discovered in 1973, is an endopeptidase involved in the removal of angiotensin II found in blood vessels, the heart, and the proximal renal tubule [128]. Its inhibition eventually results in vasodilation, natriuresis, diuresis, and further inhibition of fibrosis, but can also cause vasoconstriction, water retention, and hypertrophy [129].

Recently, the combination of an ARNI, sacubitril, with valsartan, an ARB, has been approved for symptomatic NYHA class II or III heart failure with systolic dysfunction (Table 11) [103,124].

Valsartan was initially approved for treating hypertension [134] and later for heart failure treatment, with a proven reduction in cardiovascular death [124]. This combination was proposed because of the mixed substrates of neprilysin, which have been shown to reduce blood pressure and volume, as well as increase sodium, water excretion, and vasodilation [135].

However, few studies have addressed the pediatric population, and some authors find no benefit of sacubitril–valsartan combination in patients with complex CHD [131]. Currently, there is a multicenter pediatric trial (PANORAMA-HF) that will address the possibility that the combination of sacubitril–valsartan is superior to enalapril for the treatment of pediatric heart failure with reduced systolic function [43].

#### 2.5.2. Antiarrhythmics

Antiarrhythmic drugs play a major role in treating atrial and ventricular arrhythmias, particularly for the symptomatic relief and prophylaxis of these conditions (Table 12) [136]. Specifically, they are sorted according to their mechanism of action based on the Vaughan Williams classification [137,138].

This classification remains valid to date [139]. However, the modified classification included a class 0, including drugs that act on sinoatrial automaticities, such as ivabradine, a medication used to reduce heart rate in sinus tachycardia, with or without concomitant heart failure [125].

**Table 12:** Indications, dosing regimen, and adverse effects/contraindications of antiarrhythmics.

Drug for CHD	Indication	Dosing Regimen	Adverse Effects/ Contraindications	Refs.
Antiarrhythmics	Atrial fibrillation rate and rhythm control, supraventricular tachycardia in adults with CHD, ventricular arrhythmias, and Wolff–Parkinson–White syndrome	-Class Ia: Procainamide: 500–1250 mg q6h oral; 15 mg/kg IV -Class Ib: Mexiletine: 150–250 mg q8h oral -Class Ic: Flecainide: 50–150 mg q12h oral -Class III: Amiodarone: ≤200 mg/d Sotalol: Initial: 80 mg q12h Increase to 160 mg q12h (max 320 mg) oral -Class IV: Diltiazem: 1.5–2 to 3–5 mg/kg/d	-QT prolongation: class I, III, and IV -Torsades de pointes: class IV -Contraindicated in structural disease: quinidine (class Ia), propafenone, and flecainide (class Ic)	[103,121,140,141]

##### Class I

Sodium channel blockers represent class I, such as procainamide, and are divided into three subgroups based on the speed of dissociation from their receptor [125,138]. These antiarrhythmics are contraindicated in patients with CHDs, as class I drugs can depress ventricular function, especially in patients with decreased systolic ejection fraction [142].

Specifically, these agents have the risk of causing proarrhythmic events and they increase the risk of ventricular arrhythmias in patients with tetralogy of Fallot [141]. Nonetheless, other authors claim that class Ic drugs can be used in patients with simple CHDs, with no ventricular incisions or patches, no ventricular hypertrophy, no coronary artery disease, and preserved ventricular function (i.e., atrial septal defect) [143].

##### Class II

Beta-blockers, such as propranolol, constitute the second class and exert their action by reducing heart rate and conduction velocity, and increasing the duration of the effective refractory period [35]. Given their anti-adrenergic effects on the sinoatrial and atrioventricular node, beta-blockers can be used for supraventricular and ventricular tachycardias, node reentrant tachycardias, and atrioventricular reentrant tachycardia [141].

Furthermore, there is a class IIa recommendation to use beta-blockers, such as bisoprolol or metoprolol, for the acute and long-term management of supraventricular arrhythmias in adult patients with CHDs [144]. In this regard, choosing a specific beta-blocker is important, patients with asthma should be prescribed a β_1_-selective blocker (atenolol, esmolol, or metoprolol), patients with coexisting hypertension should use an alpha and beta-blocker (labetalol or carvedilol), and patients with liver dysfunction should use renally excreted blockers (atenolol or nadolol) [141].

##### Class III

Potassium channel blockers encompass class III, which includes sotalol, ibutilide, dofetilide, and amiodarone, one of the most effective drugs in the prevention and control of supraventricular tachycardias and ventricular tachyarrhythmias in CHD [121,143]. Amiodarone has been successfully used since 1960 and is effective at controlling postoperative incessant atrial arrhythmias and arrhythmias associated with structural defects, but carries a high risk for long-term toxicity, such as pulmonary fibrosis, hepatic dysfunction, and thyroid abnormalities [142].

Comparatively, sotalol, a methanesulphonanilide that has a dual delayed rectifier potassium current and beta-adrenergic-blocking activities [145], has shown safe and effective properties for the acute termination and maintenance therapy of supraventricular tachycardias resistant to adenosine and ventricular tachycardias in children with or without CHDs [146]. Nonetheless, other studies have shown high rates of proarrhythmic events and an increase in all-cause mortality [141].

Moreover, in a multicenter retrospective study, dofetilide demonstrated effective initial suppression of atrial fibrillation in 85% of patients with CHDs [142]. Studies have shown that class III antiarrhythmics have been associated with a lower risk of atrial arrhythmia recurrence when compared to other classes in patients with CHDs [147].

##### Class IV

Class IV includes nondihydropyridine calcium channel blockers, which are mainly used in CHDs for atrial tachycardia and fibrillation, as well as atrioventricular blockade [148]. Specifically, there is a class IIa recommendation for the usage of either verapamil or diltiazem for acute treatment, long-term management, and rate control of supraventricular arrhythmias in adult patients with CHDs [144].

Moreover, these calcium channel blockers can be used for SA and AV node-dependent arrhythmias, multifocal atrial tachycardia, and ventricular tachyarrhythmias involving the Purkinje fibers (fascicular or Belhassen ventricular tachycardia) [141].

##### Other Relevant Classes

Recently, the newly updated classification included the mechanosensitive channel blockers (class V) that block transient receptor potential channels (TRPC23/TRPC6) involved in intracellular calcium signaling, with a drug currently under investigation, N-(p-amylcinnamoyl) anthranilic acid [125].

Additionally, class VI was proposed as drugs that target the electrotonic coupling between cells, such as the ionic late inward sodium and L-type calcium channels, with two prototype drugs: roscovitine (reduces pedestal current) and gabapentinoids (shift the steady-state activation towards the depolarizing direction) [149].

Finally, the last class added (class VII) involves drugs that exert long-term effects on arrhythmic tendencies through the modification of structural remodeling and include ACEIs, ARBs, statins, and omega-3 fatty acids [125].

#### 2.5.3. Digoxin

Derived from a perennial herb, digoxin was identified in Western medicine in 1930 [150]. Though it was traditionally recommended for pediatric heart failure [122], digoxin is currently recommended for the symptomatic management of patients with atrial fibrillation and flutter, as well as congestive heart failure [126]. It inhibits the Na^+^/K^+^-ATPase pump of the heart (Figure 1), causing an increase in a parasympathetic tone that blocks the sinoatrial and atrioventricular nodes [151]. Digoxin is excreted renally and is available in both oral and intravenous forms [141].

Digoxin also increases cardiac inotropism and intracellular calcium [141]. In this regard, it has been hypothesized that its treatment results in improved interstage survival in patients without prior arrhythmia [152], particularly for those who have had previous stage-1 palliation of single-ventricle disease [153]. Regarding its role as an antiarrhythmic, digoxin can potentially terminate SA and AV node-dependent arrhythmias and can slow down supraventricular tachycardias [141]. Recent advances in prenatal diagnosis have increased the possibilities of applying transplacental treatments, with studies showing improvements in heart failure in patients with CHD (Table 13) [154].

Additionally, digoxin treatment may be associated with increased survival in patients who underwent Damus–Kaye–Stansel or Norwood procedures during the interstage period, but it has not shown a benefit in patients with single-ventricle physiology during this period [157].

#### 2.5.4. Non-Steroidal Anti-Inflammatory Drugs

Since salicylate was first isolated in the 1830s, NSAIDs have been one of the most prescribed drugs worldwide [158]. Among them, indomethacin has been used for treating patent ductus arteriosus since the 1970s, and ibuprofen was also approved for the closure of patent ductus arteriosus in 2006 (Table 14) [123].

Currently, ibuprofen and indomethacin remain approved for treating patent ductus arteriosus in the pediatric population [127]. Furthermore, the application of early treatment (<12 h of age) has been associated with a reduction in pulmonary and periventricular or intraventricular hemorrhage, all associated with worse outcomes [165].

Since ibuprofen and indomethacin have potential adverse effects on vascular and organ perfusion [166,167], oral or intravenous administration of acetaminophen has been proposed due to its high rate of patent ductus arteriosus closure with minimal adverse effects [168]. It is especially recommended for patients with contraindications to ibuprofen management, treatment failure, or initial treatment [167].

## 3. Recent Clinical Trials Testing Drugs for CHD Treatment

Clinical trials addressing heart disease are not found in the level of interest that research demands, as only nearly 7% of over 5000 clinical trials are currently ongoing [169]. Moreover, CHD is heterogeneous and has endured as a therapeutic desert in contrast to cardiovascular disease contracted during adulthood [170,171]. Thus, clinical trials are required to assess the effects of novel drugs, along with their corresponding dosing schedule, particularly during childhood [172].

Despite having a robust work hypothesis, designing a clinical trial with CHD patients may be difficult [170]. Trials with children do have not both the frequency and ease that could be expected, especially when randomized [173]. Counterintuitively, research on the safety and efficacy of drugs for adult CHD remains limited [174]. For instance, the study of Woudstra et al. was the first large assessment of polypharmacy associations with clinical outcomes in adult CHD, despite its self-claimed limitations such as data unavailability for over-the-counter medication [175].

Likewise, drawbacks usually arise in prospective studies, such as considering subgroups of CHD patients with certain defects or being terminated before scheduled due to a lack of enrollment [43,176]. Nevertheless, large randomized double-blind trials assessing the effects of candidate drugs and comparing their results with previously established molecules are of high interest for clinical research [116].

In this regard, Zaragoza-Macias et al. have indicated that there is no conclusive evidence regarding the beneficial effect of therapy on adult patients with systemic right ventricle dysfunction; thus, randomized or comparative trials are needed to determine the efficacy of drugs such as ACEIs, ARBs, and beta-blockers for such specific conditions [20]. Interestingly, a clinical trial is studying the effectiveness of adding beta-blockers to the background therapy of pulmonary arterial hypertension, as well as two randomized clinical trials evaluating the effects of spironolactone monotherapy or sequential therapy to ambrisentan [176].

In a study by Durongpisitkul et al., pulmonary arterial hypertension derived from CHD has shown intermediate-term benefits after the treatment of generic bosentan as a complementary therapy to sildenafil, with a significant improvement in the scores of low-risk criteria after one year [98]. Additionally, a study by McLaughlin et al. assessing the safety and clinical outcomes after the treatment with macitentan in this same CHD population indicated an important number of patients (4268) in follow-up [177]. Furthermore, Iwasawa et al. have indicated that pulmonary toxicity induced by amiodarone demands future prospective studies in younger patients, considering also their drawbacks, such as a small sample size and study type [121].

The need for efficacy and safety trials in the pediatric population with CHDs is further emphasized by a recent study conducted by Meliota et al., showing that 85% of cardiovascular drugs are used off-label and more than 88.3% of patients received more than one off-label drug, thus increasing the risk for adverse effects and unexpected outcomes [178]. Recently, Diller et al. proposed the inclusion of new knowledge from genetics, genomics, and the environmental impact on disease expression and patient outcomes, as well as the introduction of machine learning to improve information collected throughout the lifetime of patients with CHD [179].

Lastly, a review of randomized controlled trials conducted by Hummel et al. in patients younger than 5 years demonstrated that the use of levosimendan, a calcium sensitizer, did not show any significant differences in the prevention of low cardiac output syndrome in patients with CHD undergoing surgery when compared to standard inotrope treatments [180]. In 2019, a phase II/III multicenter study was launched to analyze the age-appropriate dose recommendation, metabolomics, and pharmacogenetics of enalapril in children with heart failure due to dilated cardiomyopathy or CHD [181]. In this regard, a recent study demonstrated that the physiological age-appropriate dose based on pharmacokinetics ranged from 0.25 to 16 mg/d and the mean body weight dose ranged from 0.06 to 0.27 mg/kg [182].

## 4. Discussion

CHD represents a complex spectrum of diseases continuously treated with a variety of novel therapies, which have a variable impact on the lives of patients [183]. Historically, most therapeutic interventions have been empirical, as CHDs are time-sensitive and progressive for patients, thus requiring life-prolonging or life-saving treatments [15].

Specifically, it is estimated that 4–28% of patients with CHD will eventually develop one of four types of pulmonary arterial hypertension: (1) Eisenmenger syndrome, (2) left-to-right shunts, (3) pulmonary arterial hypertension with coincidental CHD, and (4) persistent/recurrent pulmonary arterial hypertension after correction of CHD [119]. This ever-growing population posed new challenges for a multidisciplinary team to achieve optimal care [184].

Studies have shown that drugs, such as angiotensin receptor blockers [124]; prostaglandins [11]; and ACE [13,14], endothelin-1 [99], and PDE-5 inhibitors [12], have a positive impact on mortality and quality of life in patients with CHD. Likewise, treatments, such as riociguat [120], macitentan [98], and a combination of bosentan with sildenafil [94], have given positive results, but studies in pediatric patients are needed.

Naturally, the use and study of analyzed drugs in this review demand accurate clinical trials. A trend analysis of NIH-funded clinical trials addressing CHD showed that nearly less than 0.45% of the studies were aimed at the pediatric population, compared with the remaining 99.95% of clinical trials for general cardiovascular disease [185]. In terms of the current challenges of clinical trials, we concur on maximizing the study of cohorts [186], increasing the sample size regardless of the management issues of younglings [170], and enrolling heterogeneous patients, thus avoiding any neglection of participants, e.g., trisomy-21 patients [187].

Authors such as Hill et al. have highlighted the role of clinical trial simulations for de novo design [173], which can be helpful for CHD studies. In this regard, Cedars and Kutty support that trials may need a different approach to achieve success, although there are certain large sound clinical trials promoting progress for CHD [170]. This results are significant in confirming the effect of ACEIs, ARBs, and beta-blockers through large prospective randomized trials [20]; establishing the efficiency of bosentan in a defined period to reduce its common adverse effects on patients with pulmonary arterial hypertension [187]; and determining the optimal dose and timing for the initiation of ethacrynic acid treatment, a drug which has reportedly performed better than furosemide [188].

Additionally, new drugs should be developed to target specific genomic characterizations and variations in the RAAS or adrenergic signaling pathways to better improve responses to treatment and eventually ventricular function and survival of patients with CHD [189]. Likewise, a personalized approach should be sought, from prenatal screening to planning during infancy and childhood, and an eventual transition to adulthood with a multidisciplinary combination of interventions, including surgical, pharmacological, and percutaneous options [190], consistent with the aim to boost the impact of investment on health-directed CHD research [185].

We acknowledge that future works in the field of CHD treatment should be specially focused on clinical trials addressing relevant CHDs, e.g., pulmonary arterial hypertension, in both pediatric and adult patients, namely with meta-analysis and systematic reviews.

## 5. Conclusions

CHD depends on various therapeutic interventions, which ultimately lead to definitive surgical correction. Although many patients are reaching adulthood, additional treatment options will improve their quality of life, especially in developing countries. Likewise, innovation should be sought for new drug candidates that address the physiological nature of CHD defects and eventual increase in survival.

## Figures and Tables

**Table 1:** Indications, dosing regimen, and adverse effects/contraindications of beta-blockers.

Drug for CHD	Indication	Dosing Regimen	Adverse Effects/ Contraindications	Refs.
Beta-blockers	Left ventricle systolic dysfunction	1st-generation: Propranolol: 4 mg/kg/d 2nd-generation: Bisoprolol 0.1–0.2 mg/kg/d 3rd generation: Carvedilol: Patients within: 28 d-23 m: 3 mg/kg 2–11 y: 2 mg/kg 12–15 y: 1 mg/kg	-Lightheadedness and dizziness -Contraindicated in asthma -Hypoglycemia in infants with sotalol use	[27,28,29,30,31,32]

**Table 2:** Indications, dosing regimen, and adverse effects/contraindications of ACEIs for CHD.

RAAS Inhibitor for CHD	Indications	Dosing Regimen	Adverse Effects/ Contraindications	Refs.
Angiotensin-converting enzyme inhibitors (ACEIs)	Asymptomatic CHDs and symptomatic heart failure	-Captopril: Neonates: 0.4–1.6 mg/kg/d in 3 doses Infants: 0.5–4 mg/kg/d in 3 doses -Enalapril: Children > 2 y: 0.1–0.5 mg/kg/d in two doses -Lisinopril: 5 mg/d	-Acute kidney injury, angioedema, cough, hyperkalemia, and hypotension -Contraindicated in bilateral renal artery stenosis	[14,46,47,48,49]

**Table 3:** Indications, dosing regimen, and adverse effects/contraindications of ARBs for CHD.

RAAS Inhibitor for CHD	Indications	Dosing Regimen	Adverse Effects/ Contraindications	Refs.
Angiotensin receptor blockers (ARBs)	-Left ventricle systolic dysfunction in patients with intolerance to ACEIs -Slows the progression of genetically triggered aortopathy disease	Losartan: 25–50 mg/d Valsartan: 1.3 mg/kg/d	Acute kidney injury, diarrhea, dizziness, headache, hyperkalemia, and hypotension	[24,49,54,55,56]

**Table 4:** Indications, dosing regimen, and adverse effects/contraindications of loop diuretics.

Diuretic for CHD	Indication	Dosing Regimen	Adverse Effects/ Contraindications	Refs.
Loop Diuretics	-Decompensated heart failure -Fluid overload in CHD	Furosemide: 0.08 mg/kg/h	Hypercalciuria, nephrolithiasis, osteoporosis, and pre-renal azotemia Tolerance after chronic use	[14,65,66,67,68]

**Table 5:** Indications, dosing regimen, and adverse effects/contraindications of thiazide diuretics.

Diuretic for CHD	Indication	Dosing Regimen	Adverse Effects/ Contraindications	Refs.
Thiazide Diuretics	Postoperative fluid overload	-Chlorothiazide: 10 mg/kg/d -Hydro- chlorothiazide: 1–2 mg/kg/d	-Hyperglycemia, hyperlipidemia, hyperuricemia, hypokalemia, metabolic alkalosis, and prerenal azotemia -Contraindicated in patients with anuria	[26,49]

**Table 6:** Indications, dosing regimen, and adverse effects/contraindications of potassium-sparing diuretics for the treatment of CHD.

Diuretic for CHD	Indication	Dosing Regimen	Adverse Effects/ Contraindications	Refs.
Potassium -Sparing Diuretics	-Symptomatic heart failure, systemic right ventricle morphology, double-inlet right morphology ventricle, hypoplastic left heart syndrome, and transposition of great vessels with arterial switch operation repair	-Spironolactone: 25–75 mg/d -Eplerenone: 50 mg/d	-Anti-androgenic and estrogenic effects, gynecomastia, and hyperkalemia	[54,73,74]

**Table 7:** Indications, dosing regimen, and adverse effects/contraindications of ERAs.

Vasodilator for CHD	Indication	Dosing Regimen	Adverse Effects/ Contraindications	Refs.
Endothelin-1 Receptor Antagonists (ERAs)	-Adult pulmonary arterial hypertension associated with CHD -Idiopathic pulmonary hypertension -Eisenmenger syndrome	Bosentan: 2 mg/kg q12h	Dizziness, flushing, hemoptysis, increased LFTs, and non-sustained ventricular tachycardia	[89,90,91,92]

**Table 8:** Indications, dosing regimen, and adverse effects/contraindications of PDE-5 inhibitors.

Vasodilator for CHD	Indication	Dosing Regimen	Adverse Effects/ Contraindications	Refs.
PDE-5 Inhibitors	Pulmonary arterial hypertension and pulmonary hyper flow from any CHD	Sildenafil: 1 mg/kg q8h	Dizziness, lupus-like syndrome, orthostatic hypotension, peripheral edema, and refle Xtachycardia	[49,102,104]

**Table 9:** Indications, dosing regimen, and adverse effects/contraindications of prostaglandins.

Vasodilator for CHD	Indication	Dosing Regimen	Adverse Effects/ Contraindications	Refs.
Prostaglandins	Aortic, mitral, pulmonary, and tricuspid atresia, aortic stenosis, interrupted aortic arch, hypoplastic left heart syndrome, pulmonary stenosis, severe mitral stenosis, and transposition of great vessels with intact interventricular septum	PGE1: Initial dose of 0.025 µg/kg/min to 0.01 µg/kg/min	Apnea (dose-dependent), bradycardia, diarrhea, disseminated intravascular coagulation, fever, hypotension, hypothermia, and seizures	[108,109,110,111,112,113]

**Table 10:** Indications, dosing regimen, and adverse effects/contraindications of sGC stimulators.

Vasodilator for CHD	Indication	Dosing Regimen	Adverse Effects/ Contraindications	Refs.
Stimulators of soluble guanylate cyclase (sCG)	Adult pulmonary arterial hypertension associated with CHD	Riociguat: 1.5–2.5 mg q8h	-Diarrhea, dizziness, dyspepsia, headache, hypertension, nausea, peripheral edema, and vomiting -Contraindicated during pregnancy	[117,118,119]

**Table 11:** Indications, dosing regimen, and adverse effects/contraindications of ARNIs for CHD.

Drug for CHD	Indication	Dosing Regimen	Adverse Effects/ Contraindications	Refs.
Angiotensin Receptor Neprilysin Inhibitors (ARNIs)	-Symptomatic NYHA class II or III -Heart failure with systolic dysfunction	Sacubitril–valsartan: 3.1 mg/kg q12h	Renal dysfunction	[103,130,131,132,133]

**Table 13:** Indications, dosing regimen, and adverse effects/contraindications of digoxin for CHD.

Drug for CHD	Indication	Dosing Regimen	Adverse Effects/ Contraindications	Refs.
Digoxin	-Symptomatic heart failure -Adult and fetal tachyarrhythmias	Digoxin: 8–10 mcg/kg/24 h in children from 2 to 10 years	Atrial tachycardia, complete heart block, delirium nausea, hypomagnesemia, hypokalemia, sinoatrial/atrioventricular junction, sinus arrest, vomiting, and visual changes	[141,155,156,157]

**Table 14:** Indications, dosing regimen, and adverse effects/contraindications of NSAIDs for CHD.

Drug for CHD	Indication	Dosing Regimen	Adverse Effects/ Contraindications	Refs.
Non- steroidal anti- inflammatory Drugs (NSAIDs)	-Patent ductus arteriosus closure in preterm infants	-Ibuprofen (3 doses): 10–5–5 mg/kg/d -Indomethacin (3–6 doses): 0.2 mg/kg IV -Acetaminophen (3–7 d): 15 mg/kg q6h	Gastrointestinal and renal toxicity, heart failure exacerbation, and hypertension	[159,160,161,162,163,164]

## Data Availability

Not applicable.

## Supplementary Materials

The following supporting information can be downloaded at: https://www.mdpi.com/article/10.3390/jcdd9070201/s1. Figure S1. Flow diagram for literature review inspired by PRISMA guidelines.
