# Supplementary figures and images for: Congenital Heart Disease: The State-of-the-Art on Its Pharmacological Therapeutics
Source: J Cardiovasc Dev Dis. 2022 Jun 26;9(7):201. doi: 10.3390/jcdd9070201 (PMC9316572; doi:10.3390/jcdd9070201)

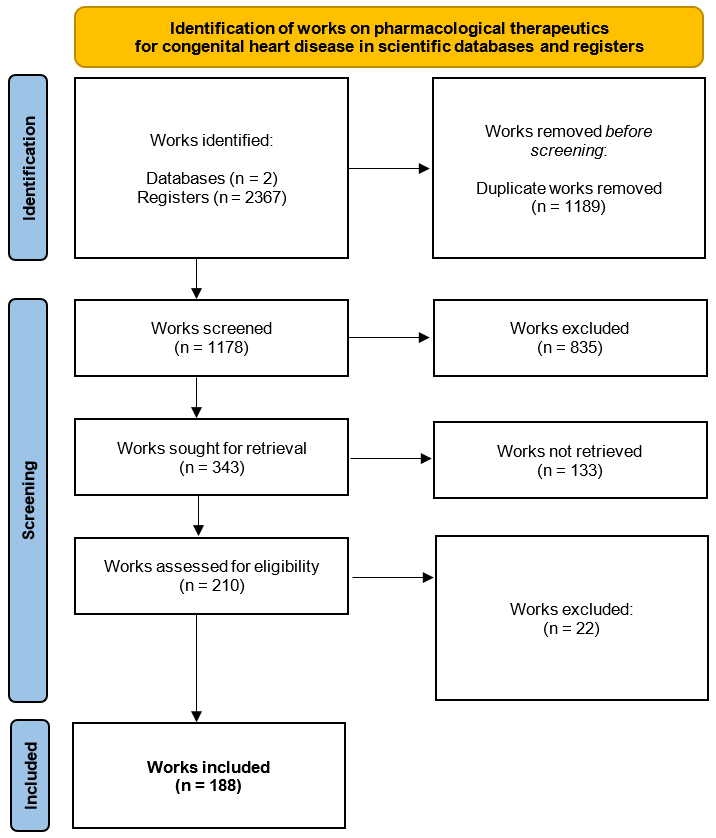

Supplement: Supplementary file 1 [file jcdd-09-00201-s001.zip › jcdd-1748574-Supplementary Figure S1.PNG]
